# Brain Metastases in Lung Cancers with Emerging Targetable Fusion Drivers

**DOI:** 10.3390/ijms21041416

**Published:** 2020-02-19

**Authors:** Aaron C. Tan, Malinda Itchins, Mustafa Khasraw

**Affiliations:** 1 Division of Medical Oncology, National Cancer Centre Singapore, Singapore 169610, Singapore; 2 Department of Medical Oncology, Royal North Shore Hospital, St Leonards, NSW 2065, Australia; malinda.itchins@sydney.edu.au; 3 Northern Clinical School, Faculty of Medicine and Health, University of Sydney, St Leonards, NSW 2065, Australia; 4 The Preston Robert Tisch Brain Tumor Center, Duke Cancer Institute, Duke University, Durham, NC 27708, USA; mustafa.khasraw@duke.edu

**Keywords:** brain metastases, fusion drivers, non-small cell lung cancer, targeted therapy

## Abstract

The management of non-small cell lung cancer (NSCLC) has transformed with the discovery of therapeutically tractable oncogenic drivers. In addition to activating driver mutations, gene fusions or rearrangements form a unique sub-class, with anaplastic lymphoma kinase (*ALK*) and c-ros oncogene 1 (*ROS1*) targeted agents approved as the standard of care in the first-line setting for advanced disease. There are a number of emerging fusion drivers, however, including neurotrophin kinase (*NTRK*), rearrangement during transfection (*RET*), and neuregulin 1 (*NRG1*) for which there are evolving high-impact systemic treatment options. Brain metastases are highly prevalent in NSCLC patients, with molecularly selected populations such as epidermal growth factor receptor (*EGFR*) mutant and *ALK*-rearranged tumors particularly brain tropic. Accordingly, there exists a substantial body of research pertaining to the understanding of brain metastases in such populations. Little is known, however, on the molecular mechanisms of brain metastases in those with other targetable fusion drivers in NSCLC. This review encompasses key areas including the biological underpinnings of brain metastases in fusion-driven lung cancers, the intracranial efficacy of novel systemic therapies, and future directions required to optimize the control and prevention of brain metastases.

## 1. Introduction

Non-small cell lung cancer (NSCLC) is a leading cause of cancer death globally [1] and is characterized by a high prevalence of brain metastases (BM) of up to 50% during the course of the disease [2,3]. The presence of BM portends a poor prognosis, both in terms of survival and response to systemic therapies, and has associated morbidity, including from the side effects of treatment [4]. Currently, the treatment paradigm consists of surgery, radiotherapy, systemic therapy, and palliative care support, and in those with advanced disease, the appropriate sequence or combination of local and systemic therapies is crucial. However, with rapidly evolving systemic treatment options including molecular therapies and immunotherapy, patients with BM remain a patient population of special interest. Novel therapies may have variable intracranial efficacy, and typically only patients with stable asymptomatic BM are enrolled in clinical trials or may be excluded altogether, particularly in the setting of leptomeningeal carcinomatosis.

Our understanding of NSCLC has transformed with the discovery of molecular subtypes and therapeutically targetable oncogenic drivers, such as epidermal growth factor receptor (*EGFR*) activating mutations, anaplastic lymphoma kinase (*ALK*), and c-ros oncogene 1 (*ROS1*) gene fusions/rearrangements [5,6,7]. Tyrosine kinase inhibitor (TKI) therapy is now standard of care in the first-line setting for advanced disease in patients that harbor such alterations. Accordingly, there exists a substantial and growing body of research pertaining to the understanding of BM in these populations [8]. However, there are a number of emerging fusion drivers, including neuregulin 1 (*NRG1*), neurotrophin kinase (*NTRK*), and rearrangement during transfection (*RET*), for which there are evolving systemic treatment options. Indeed, driver gene fusions in cancer are more common than previously thought [9]. Much less is known on the molecular mechanisms of BM in lung cancers with emerging targetable fusion drivers. This review will encompass key areas including the biological underpinnings of BM in lung cancers harboring an oncogenic fusion driver, the intracranial efficacy of novel systemic therapies, and future directions to optimize the control and prevention of BM.

## 2. Molecular Understanding of BM in NSCLC with Fusions Drivers

The overall incidence of BM in advanced NSCLC patients at diagnosis has been estimated at 10–20% [3,10]. This is higher in *EGFR* mutant NSCLC at 23–32% [11,12], and in *KRAS* mutant NSCLC at 29% [13]. The incidence may also be higher in patients with *ALK* fusions at 20–39% [14,15,16], and in patients with *ROS1* fusions at 19–36% [15,16]. The underlying molecular biology behind this increased incidence of BM in fusion-driven NSCLC remains to be elucidated. Significant progress has been made, however, in our understanding of the mechanisms behind the development of BM in general. A sequential stepwise process may occur, incorporating the extravasation and invasion of cancer cells across the blood–brain barrier (BBB), interaction with the brain microenvironment, vascular co-option, and dormancy, and finally angiogenesis and proliferation [17,18,19]. An enhanced understanding of this process is crucial to the development of more effective therapies and preventative strategies.

The BBB is of particular importance in tumors treated with small-molecule TKIs. Due to the BBB, the brain has long been considered a ‘sanctuary site’, preventing the ability of drugs to penetrate the central nervous system (CNS) [20,21]. Moreover, present are active exclusion mechanisms such as efflux pump(s) glycoprotein P (P-gp) and breast cancer resistance protein (BCRP) [22]. Consequently, this may result in both the poor intracranial efficacy for many systemic therapies including many TKIs and the brain as a common site of disease progression after treatment failure of systemic therapy. While the BBB may have increased permeability in the presence of BM, achieving sufficient drug concentrations for anti-tumor efficacy remains a challenge [23]. Other brain tissue microenvironment factors may also be crucial in determining response to therapy [24]. Especially for *EGFR* mutant and *ALK*-rearranged NSCLC, however, many newer generation TKIs have been engineered specifically to have greater CNS penetrability and resultant superior intracranial efficacy compared to first-generation TKIs [25,26,27]. Translational and preclinical studies have supported the greater intracranial penetration for these newer agents [28,29].

There have been numerous studies investigating potential biomarkers or predictors for the development of BM in NSCLC. One such study by Wang et al. [30] performed panel-based next-generation sequencing (NGS) of 61 paired primary and resected BM tumors. They found alterations of genes related to cell cycle regulation and phosphatidylinositol 3-kinase (PI3K) pathway signaling were enriched and tumor mutation burden (TMB) was increased in the resected BM compared to the paired primary lung tumors. Another study that molecularly profiled 3424 unmatched NSCLC tumors found that lung adenocarcinoma BM had the greatest median TMB compared to other extracranial metastatic sites [31]. Together, this potentially reflects the genetic divergence and evolution of BM compared to other metastatic sites. In fusion-driven NSCLC, however, and fusion driven cancers in general, TMB is generally low [32]. This suggests tumor evolution cannot fully explain the propensity for BM in fusion-driven NSCLC. Importantly, however, many oncogenic kinases within driver fusions such as *ALK* and *RET* are known to constitutively activate the PI3K/Akt/ mechanistic target of rapamycin (mTOR) pathway, including in response to treatment, resulting in tumorigenesis [33,34,35]. This implicates the PI3K/Akt/mTOR pathway as a mechanism by which fusion-driven NSCLC develop BM. *LPCAT1* gene expression may play a key role in promoting BM via the PI3K pathway [36]. Furthermore, prior studies of BM in breast cancer and melanoma have also illustrated the importance of PI3K pathway upregulation [37,38,39]. Finally, the specific fusion partner may also play a role, potentially due to differing protein conformations. *CD74*-*ROS1* fusions, for example, have been demonstrated to have higher rates of BM compared to other *ROS1* fusion partners [40].

## 3. *RET* Fusion-Positive NSCLC

*RET* fusions were first identified in NSCLC in 2012 by four independent groups and occur in approximately 1–2% of NSCLC patients [41,42]. The incidence of BM in *RET* fusion-positive NSCLC at diagnosis has been estimated at 25% based on a combined multi-institutional registry and bi-institutional dataset cohort of 133 patients [43]. In this retrospective study, the lifetime prevalence of BM was 46%. Multikinase inhibitors, such as cabozantinib and vandetanib, have been demonstrated to have limited systemic efficacy in phase II clinical trials [44,45,46]. Accordingly, intracranial efficacy has also been seen to be limited. In the phase II trial of cabozantinib for *RET* fusion-positive NSCLC, two of five patients with untreated baseline BM had intracranial disease control, while 10% of patients discontinued cabozantinib due to the development or progression of BM [44]. In the aforementioned retrospective study by Drilon et al. [43], intracranial response was seen in only two of 11 (18%) patients that were treated with a multikinase RET TKI, with a median overall progression-free survival (PFS) of 3.9 months. There have also been limited reports of intracranial activity for alectinib [47,48].

Subsequently, however, more selective and potent RET TKIs have been developed, notably selpercatinib (LOXO-292) and pralsetinib (BLU-667). Early efficacy data from phase I/II clinical trials have illustrated impressive and durable responses in patients with *RET* fusion-positive NSCLC [49,50]. Similarly, there is evidence of much greater intracranial efficacy for selpercatinib and pralsetinib compared to the previous multikinase TKIs [50,51]. In the LIBRETTO-001 study, durable intracranial responses to selpercatinib were seen in ten of 11 (91%) patients with measurable intracranial disease, with disease control in additional patients with non-measurable BM [49,51]. Significant intracranial activity was also seen with pralsetinib on the ARROW study, in which 39% had baseline BM [50]. Shrinkage with pralsetinib was seen in 7/9 (78%) patients with measurable BM. Furthermore, there have been documented responses to selpercatinib in patients with leptomeningeal disease and in various contexts such as intracranial progression after prior systemic and local therapies [48,52]. There is also preclinical evidence that demonstrates the enhanced intracranial efficacy of these selective TKIs. In an orthotopic mouse tumor model, *CCDC6-RET* fusion-positive patient-derived xenograft (PDX) cell suspensions were injected intracranially, and mice were treated with selpercatinib or ponatinib (a multikinase TKI) [48]. Selpercatinib significantly prolonged survival compared with vehicle- or ponatinib-treated mice.

Interestingly, on a phase I trial of vandetinib in combination with everolimus, intracranial response was seen in a patient with *RET* fusion-positive and *AKT2* gene amplified NSCLC [53]. The addition of an mTOR inhibitor was proposed to improve the intracranial efficacy of vandetinib by increasing BBB penetration via modulating P-gp- and BCRP1-mediated efflux, and inhibition of AKT2, a critical component of the PI3K/mTOR pathway. Oncogenic *RET* fusions have been demonstrated to activate the kinase domain, leading to uncontrolled activation of the PI3K pathway [54]—again implicating this pathway as a potential key driver of BM in *RET* fusion-positive NSCLC.

## 4. *NTRK* Fusion-Positive NSCLC

*NTRK* fusions consist of gene rearrangements involving *NTRK1*, *NTRK2*, and *NTRK3*, which result in constitutive activation of the fusion protein containing the tropomyosin receptor tyrosine kinase (TRK) kinase domain [55]. The incidence of *NTRK* fusions in NSCLC is approximately only 0.1–1% [55,56,57]. However, they have been identified ubiquitously in rare cancers such as secretory carcinomas of the breast and salivary gland [58] and infantile fibrosarcoma [59], and also at low frequencies across a wide range of common cancers [60]. The exact incidence of BM in *NTRK* fusion-positive NSCLC is unknown, although, in one retrospective series of 4872 NSCLC patients, 4 of 8 (50%) *NTRK* fusion-positive patients had BM at diagnosis of advanced disease [57]. *NTRK* fusions are also seen in other tumor types with a high frequency of BM such as breast cancers [61]. Importantly, *NTRK* fusions are also found in primary CNS malignancies, at low frequencies in adult gliomas but much higher in up to 40% of pediatric high-grade gliomas (HGG) [62]. This may provide additional insight into the efficacy of targeted therapies for CNS disease.

There are multiple TRK inhibitors entering the clinic, most notably entrectinib and larotrectinib, with both recently receiving tumor-agnostic regulatory approvals [63]. Entrectinib is considered a multi-kinase TRK inhibitor, with additional activity against ROS1 and ALK [64]. Preliminary reports from early phase trials for entrectinib indicated intracranial response in 3/3 (100%) patients, including a NSCLC patient with 15-20 BM [65]. Updated analysis indicated an intracranial objective response rate (ORR) of 55%, with a median intracranial PFS of 14.3 months in all solid tumors [66]. In three patients with NTRK fusion-positive pediatric HGG, all had durable responses to entrectinib [67]. In the small numbers of *NTRK* fusion-positive NSCLC, entrectinib has similarly shown durable systemic and intracranial efficacy with responses in 4/6 (67%) patients [68,69]. Larotrectinib, a selective TRK inhibitor, has also been shown to be highly active in NSCLC [70]. There were initial case reports of intracranial efficacy with larotrectinib, including a NSCLC patient with a near intracranial complete response (CR) [71]. More recent analyses have also indicated intracranial response to larotrectinib in a pooled analysis of two clinical trials, with 3/5 (60%) non-primary CNS solid tumor patients achieving PR, stable disease (SD) in 1 (20%) and not evaluable in 1 (20%) [72]. This small cohort included three NSCLC patients, of which one had response. In nine primary CNS tumor patients, disease control was seen in 8/9 (89%) patients, with one (11%) not evaluable. Furthermore, the CNS was not a site of progression on larotrectinib in the initial trials across multiple tumor types in patients without baseline BM [73].

The underlying biology of BM in *NTRK* fusion-positive cancers remains to be elucidated, however, the role of TRK pathway signaling in neuronal development and differentiation may be crucial in understanding the pathogenesis [61]. *NTRK* genes are predominantly transcribed in the nervous system in adult tissues and during embryonic development [74]. Neutrophins and neutrotrophin receptors may be crucial in maintaining neuronal homeostasis [75]. Consequently, TRK proteins and activated signaling pathways may influence diverse neuronal functions, including survival and differentiation of neurons [76]. TRK fusion proteins may signal through the same downstream pathways upon neurotrophin binding as the full-length TRK proteins [77]. In cancer, tissue histology may also play a key role in determining pathway activation involved in tumorigenesis [61]. The *ETV6*-*NTRK3* fusion, for example, essentially pathognomonic in secretory breast carcinoma and infantile fibrosarcomas, results in activation of the PI3K/Akt signaling cascade and upregulation of MAPK activity and cyclin D1 expression [78]. Contrastingly, in lung cancer cell lines, although activation of PI3K and STAT3 signaling is seen, signaling via the SHC-RAS-MAPK pathway is predominant [56].

Preclinical studies confirm the ability of TRK inhibitors such as entrectinib to penetrate the BBB [64]. Furthermore, specific on-target neurologic toxicities have been seen in clinical trials due to TRK inhibition such as dizziness and cognitive changes [63]. Ultimately, greater characterization of the pathogenesis and incidence of BM in *NTRK* fusion-positive NSCLC and the intracranial efficacy of next-generation TRK inhibitors is needed, which may also elucidate our molecular understanding of BM in other *NTRK* fusion-positive cancers.

## 5. *NRG1* Fusion-Positive NSCLC

*NRG1* fusions were initially identified in 2014 in five patients with invasive mucinous adenocarcinomas (IMA) of the lung [79]. The incidence of *NRG1* fusions is estimated at 0.3% of NSCLC and 0.2% of all solid cancers based on a large series of 21,858 cases [80]. The majority of the detected *NRG1* fusions (61%), however, were found in NSCLC, with gallbladder and pancreatic cancers the next most common. Up to 61% of *NRG1* fusion-positive NSCLC is of IMA histology, although it has also been identified in both adenocarcinoma and squamous cell carcinoma [81]. *NRG1* fusions provide an extracellular anchor for the EGF domain to bind to HER3, with subsequent HER2–HER3 heterodimerization and activation of oncogenic signaling pathways including the PI3K/Akt pathway [82]. The incidence of BM in *NRG1* fusion-positive NSCLC is not well characterized, given the relative rarity of the oncogenic driver and lack of large published series of patients. The lifetime incidence of BM was only 15% in a series of 80 patients with *NRG1* fusions from a global multicenter registry [83].

Therapies targeting *NRG1* fusions in NSCLC are currently being assessed in clinical trials. Preliminary reports of a NSCLC patient with BM treated with MCLA-128, a novel bispecific HER2/3 antibody, indicated intracranial response to therapy [84]. There are also reports of systemic responses to off-label or other novel therapies with HER2/3 inhibitory activity, most notably afatinib [81]. In the limited published cases to date, none of 11 were documented to have BM at the time of commencing *NRG1* fusion-targeted therapy [81]. However, one patient with no response to afatinib developed disease progression in the brain [85]. Another patient that maintained response to afatinib for 12 months also subsequently progressed in the brain [86].

The biological mechanisms in the development of BM in *NRG1* fusion-positive NSCLC have not yet been explored. Interestingly, however, in studies of BM in unselected NSCLC, higher expression of phosphorylated HER3 has been demonstrated in BM compared to matched primary tumors [87]. Conversely, NRG1 immunohistochemistry (IHC) expression was decreased in BM versus to the primary tumors. Additionally, our understanding of BM in breast cancer may provide interesting clues. In preclinical models of BM in breast cancer, HER3 has been found to be overexpressed in BM compared to primary tumors in *HER2*-amplified and/or *PIK3CA* mutant breast tumors [88]. Neuregulins such as NRG1 and NRG2 were then found to induce in vitro resistance to PI3K inhibition via HER3 overexpression and activation. Clinical trials of PI3K/mTOR inhibitors are underway in patients with BM and PIK3CA mutations [89].

## 6. Other Rare Fusions in NSCLC

There are a number of other rare fusions that have been detected in NSCLC for which there are targeted therapies in development or approved for other indications. *FGFR* fusions were identified in approximately 0.2% of NSCLC cases from a large series of 26,054 patients and included one patient that had clinical benefit to an investigational FGFR inhibitor [90]. Similarly, low frequencies have been identified for *BRAF* fusions (0.2–2.8%) [91,92], *MET* fusions (0.04%) [93], and EGFR fusions (0.08%) [94]. Owing to the rarity of these fusion drivers and the limited epidemiological and clinical characterization of these cohorts, our understanding of the incidence of BM and intracranial efficacy of targeted therapies is limited. Undoubtedly, as methods and techniques to detect fusions continue to improve and advance, targetable fusion drivers in NSCLC may be detected with increasing frequency [95].

## 7. Future Directions

The preliminary evidence for many novel targeted therapies indicates improving intracranial efficacy compared to first-generation targeted therapies and other systemic therapies such as chemotherapy (Table 1). This may be, in part, due to the design and selection of small often macrocyclic molecule compounds with strong consideration on their ability to penetrate the BBB, with entrectinib a prominent example [96]. Nevertheless, better understanding of the biological and molecular mechanisms underlying the development and progression of BM will allow for further progress in developing new and more effective therapies for BM. Interestingly, the PI3K/Akt/mTOR pathway appears as a recurrent theme in many studies of BM, in not only NSCLC but other cancers with a proclivity for BM such as breast cancer [39] and melanoma [97]. Oncogenic activation of this pathway in fusion-driven cancers has also been commonly identified and potentially implicates this as a targetable pathway for systemic therapy against BM. Incorporating systemic therapy into the multi-modality approach for the treatment of BM is also crucial. Lessons from treatment algorithms developed for patients with more common oncogenic drivers in NSCLC, such as *EGFR* mutations and *ALK* fusions, will guide and inform the optimal approach to patients in rarer fusion subsets. Nevertheless, the use of registries and collaborative studies will be increasingly important to understand and provide insight into the efficacy of different treatments and approaches, for which prospective trials would not be feasible. The safety and efficacy of combination therapies, including local therapies, is also an area that needs to be explored further. Novel or innovative clinical trial designs, such as window-of-opportunity studies, are promising tools with which significant pathobiological knowledge of BM can be gained [17].

## 8. Conclusions

NSCLC patients with BM remain a population of unmet medical needs. The expanding number of targetable fusion drivers and the development of novel therapeutics is increasing the complexity of care for these patients. Ultimately, a greater understanding of the underlying biological and molecular mechanisms of BM will allow for more precision in the multi-modality approach to treatment in these patients.

## Figures and Tables

**Table 1:** Intracranial Efficacy of Novel Targeted Therapies in Non-small cell lung cancer (NSCLC) with Emerging Fusion Drivers.

Drug	Intracranial Efficacy	Overall Efficacy	Trial	Reference
*RET fusions*				
Selpercatinib	10/11 (91%) pts with response	ORR 68% (71/105 pts)	LIBRETTO-001	[49]
Pralsetinib	7/9 (78%) pts with response	ORR 58% (28/48 pts)	ARROW	[50]
*NTRK fusions*				
Entrectinib	4/6 (67%) pts with response	ORR 70% (7/10 pts)	ALKA-372-001, STARTRK-1, STARTRK-2	[69]
Larotrectinib	1/3 (33%) pts with response 2/3 (67%) pts with stable disease	ORR 71% (5/7 pts)	Phase 1, SCOUT, NAVIGATE	[72]
*NRG1 fusions*				
MCLA-128	Case report of response in 1 pt	-	SPP	[84]

ORR—objective response rate, Pts—patients, SPP—single patient protocol.
